# The SIBUS‐IN Fingerheld Ultrasound Machine

**DOI:** 10.1111/jocd.70313

**Published:** 2025-06-30

**Authors:** Jovian Wan, Benjamin Ascher, Leonie Schelke, Isaac Kai Jie Wong, Peter J. Velthuis, Won Lee, Hee‐Jin Kim, Kyu‐Ho Yi

**Affiliations:** ^1^ Medical Research Inc. Wonju Korea; ^2^ Think‐In Tech Paris France; ^3^ Department of Dermatology Erasmus University Medical Center Rotterdam the Netherlands; ^4^ The Artisan Clinic Singapore; ^5^ Yonsei E1 Plastic Surgery Clinic Anyang Republic of Korea; ^6^ Division in Anatomy and Developmental Biology, Department of Oral Biology Human Identification Research Institute, BK21 FOUR Project, Yonsei University College of Dentistry Seoul Korea; ^7^ You & I (Mokdong) Seoul Korea

**Keywords:** aesthetic medicine, minimally invasive procedures, real‐time imaging, ultrasound‐guided injections, vascular safety

## Abstract

**Background:**

Minimally invasive esthetic procedures risk vascular occlusion, necrosis, and other sequelae; real‐time ultrasound guidance mitigates these events, yet conventional devices are bulky and two‐handed.

**Aim:**

To present the SIBUS‐IN fingerheld ultrasound system and summarize its technical features and clinical advantages for esthetic injections.

**Methods:**

The device's specifications, workflow, and Doppler capabilities were reviewed and contrasted with published data on existing handheld scanners.

**Results:**

SIBUS‐IN mounts a 15 MHz curved transducer on the operator's index finger, streaming images wirelessly to a tablet. Motion‐gesture or voice controls allow sterile, single‐handed operation while preserving tactile feedback. Full color, power, and pulsed‐wave Doppler modes delineate vascular anatomy; images can be archived for medico‐legal documentation. Compared with Clarius, Mindray and similar units, SIBUS‐IN shortens setup time and improves access to concave or convex facial zones. Limitations include image variability in highly curved areas, infection‐control requirements, and a learning curve for novices.

**Conclusion:**

By combining ergonomic fingertip design with high‐resolution, multimode imaging, SIBUS‐IN promises greater precision, efficiency, and vascular safety in esthetic practice.

## 1 Introduction

**FIGURE 1:**
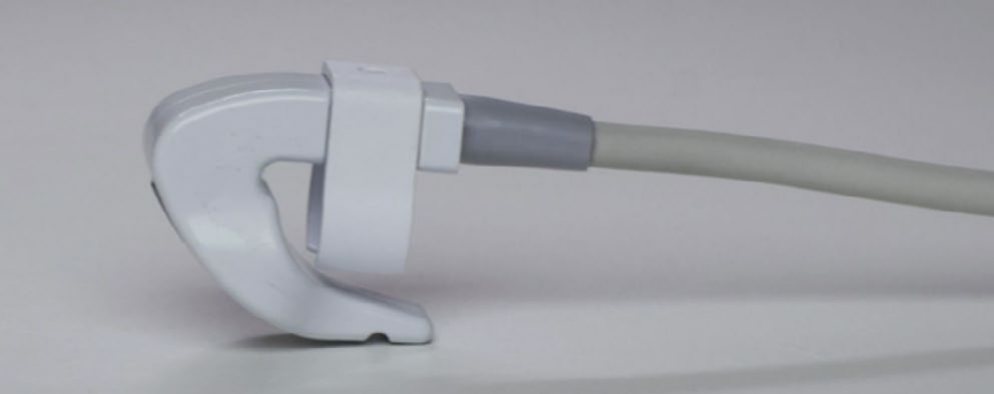
The SIBUS‐IN ultrasound probe design. The SIBUS‐IN ultrasound probe showing its compact, ergonomic design and fingertip‐mounted transducer, optimized for single‐handed operation in aesthetic procedures.

The field of minimally invasive aesthetic procedures has experienced exponential growth globally, accompanied by an increase in complications such as vascular occlusion, skin necrosis, infections, and inflammatory conditions. These challenges highlight the urgent need for innovative tools that prioritize safety, precision, and efficiency during injectable treatments [1, 2, 3]. The SIBUS‐IN (Safe Injection by Ultrasound) fingerheld ultrasound machine (Thinkin Tech SAS, France) represents a significant development (Figure 1), addressing the limitations of existing ultrasound technologies and offering healthcare professionals a compact, user‐friendly solution specifically tailored for aesthetic medicine.

The adoption of ultrasound guidance has improved the safety standards in injectable treatments. Real‐time imaging enables practitioners to visualize anatomical structures, including skin layers and blood vessels, ensuring precise needle placement and reducing risks such as vascular occlusion [4, 5, 6, 7]. While various devices from manufacturers such as Alpinion, Clarius, and Mindray offer robust imaging capabilities, these devices are primarily designed for general medical use and may present ergonomic and operational challenges in aesthetic settings [8, 9]. The SIBUS‐IN has been specifically engineered to bridge this gap by offering a solution designed with the needs of aesthetic practitioners in mind.

**FIGURE 2:**
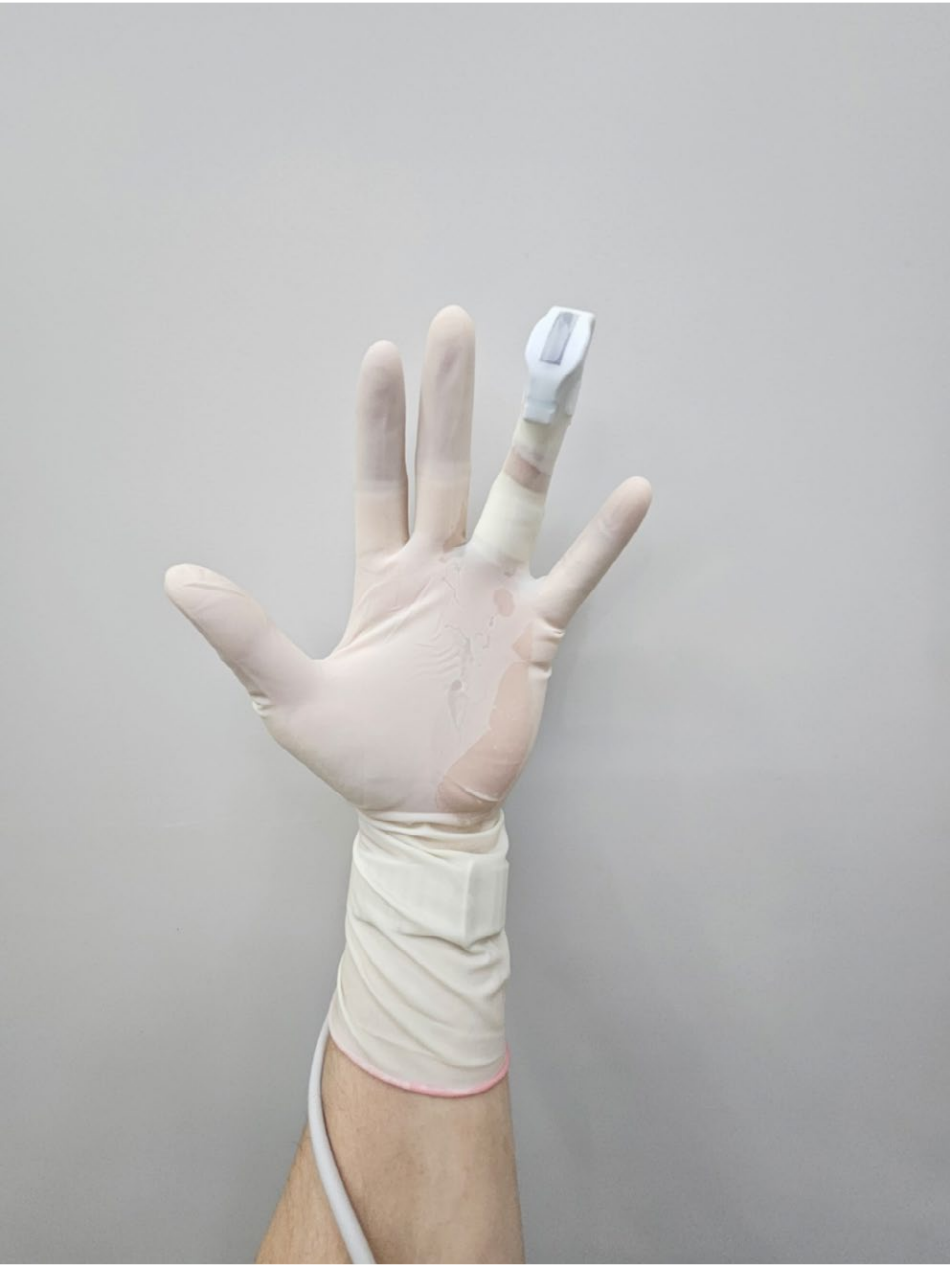
Finger position and ergonomic usage. The SIBUS‐IN probe attached to the practitioner's finger, demonstrating the ergonomic finger positioning and ease of use for precision during procedures.

Moreover, the integration of ultrasound into aesthetic practices addresses an ongoing need for real‐time guidance during injectable treatments. Complications such as vascular occlusion can result in serious adverse outcomes, including tissue necrosis and visual impairment. These risks necessitate the use of imaging tools that provide practitioners with precise anatomical insights, enabling them to navigate complex vascular structures safely and effectively [5, 10]. The SIBUS‐IN offers a compact, fingertip‐mounted device that merges traditional injection methods with modern imaging technologies, fostering improved patient outcomes and practitioner confidence (Figure 2). Previously, performing injections with real‐time visualization required assistance to manage the imaging equipment. The SIBUS‐IN enhances this workflow, enabling practitioners to independently conduct injections with simultaneous, real‐time imaging, improving procedural efficiency.

## 2 Protocol and Features of the SIBUS‐IN

The SIBUS‐IN is designed to effectively integrate into the workflow of aesthetic practitioners. Its user‐friendly interface and intuitive controls ensure a rapid learning curve, making it accessible even for those with minimal prior experience in ultrasound‐guided procedures. To optimize its use, the following protocol is recommended.

### 2.1 Preparation

1. Attach the compact curved transducer to the index finger using the ergonomic strap provided.
2. Ensure the device is wirelessly connected to the portable imaging display for real‐time feedback.
3. Activate the high‐resolution 15 MHz probe and adjust settings, including color imaging, to enhance vascular and tissue visualization.

### 2.2 Procedure

**FIGURE 3:**
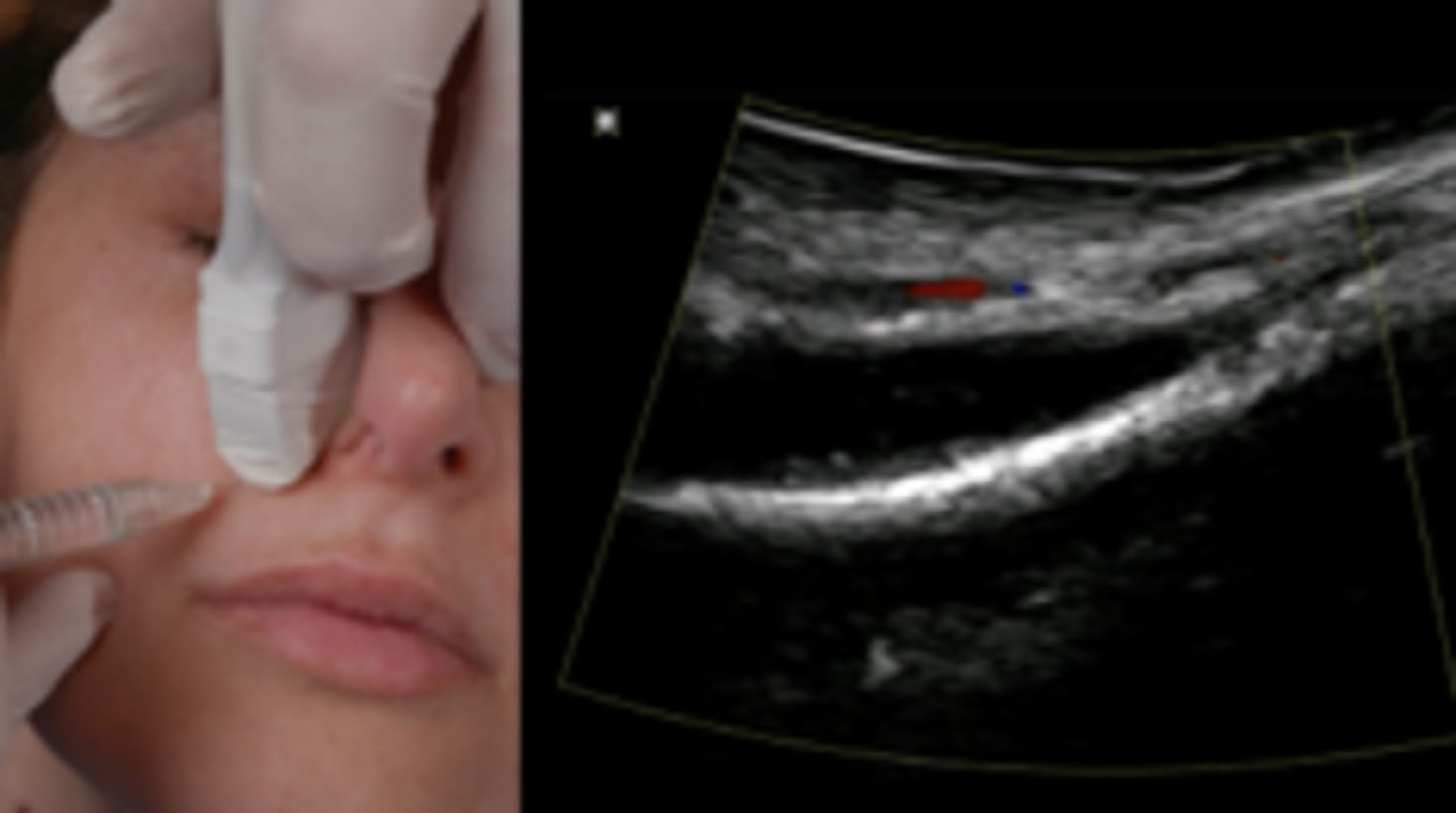
Clinical application of the SIBUS‐IN (prototype). The SIBUS‐IN in use during a live patient procedure, demonstrating real‐time imaging capabilities and ergonomic adaptability for precise injections.

1. Begin with a thorough anatomical assessment, using the SIBUS‐IN to map out critical structures, including blood vessels and skin layers.
2. Apply the transducer to the treatment area, ensuring consistent contact with the skin for optimal imaging clarity.
3. Use the device's motion gesture or voice control features to adjust imaging parameters as needed without disrupting sterility or focus.
4. Perform the injection while monitoring the needle's trajectory on the live imaging feed, navigating around high‐risk zones with precision (Figure 3, Video S1). When dissolving existing fillers, the B mode allows practitioners to pinpoint the exact location of the filler and accurately administer hyaluronidase or other agents for dissolution.

### 2.3 Post‐Procedure Documentation

1. Save imaging records directly to the patient's file for future reference and medico‐legal compliance.
2. Evaluate the post‐injection site to confirm the absence of vascular compromise or other complications.

The ergonomic design of the SIBUS‐IN ensures minimal strain during prolonged procedures. Its fingertip‐mounted transducer allows for fine, controlled movements, enabling practitioners to navigate both convex and concave facial structures with ease. Furthermore, the device's wireless functionality eliminates cumbersome cables, facilitating seamless transitions between assessment and treatment phases.

### 2.4 Key Features

The SIBUS‐IN distinguishes itself by addressing limitations commonly associated with traditional ultrasound devices. While manufacturers such as Clarius, Philips, and Mindray produce systems with high‐resolution imaging, their devices often cater to generalized medical applications, making them less suitable for dynamic and precise aesthetic procedures. The fingertip‐mounted transducer of the SIBUS‐IN offers high portability and ease of use, enabling practitioners to perform injections with greater precision and confidence.

### 2.5 Doppler Functionality

The SIBUS‐IN incorporates a comprehensive range of Doppler functionalities, including:

- Color Flow Mapping (CFM): For real‐time visualization of blood flow direction and velocity.
- Power Doppler Imaging (PDI): For enhanced sensitivity in detecting low‐speed blood flow.
- Directional Power Doppler Imaging (DPDI): To visualize both the intensity and direction of blood flow.
- Pulsed Wave Doppler (PWD): For detailed spectral analysis of blood flow at specific sites.
- B + PW (Duplex) and B + CFM/PD/DPDI + PW (Triplex): For combined imaging and Doppler analysis.

These features provide a versatile imaging solution for a variety of clinical applications, ensuring high‐quality diagnostics and enhanced safety during aesthetic procedures.

### 2.6 Wireless Functionality

The SIBUS‐IN is not entirely wireless but offers wireless functionality for data transmission between the fingertip‐mounted transducer and the portable imaging display. The probe connects to an ultrasound engine via a USB 3.0 cable, which processes the signals and transmits them to a tablet PC for real‐time visualization. This design eliminates the need for direct connections to a computer, enhancing mobility and ease of use during procedures.

### 2.7 Probe and Screen Integration

The SIBUS‐IN system consists of three main components:

1. Ultrasound Probe: A compact, high‐frequency transducer mounted on the practitioner's fingertip.
2. Ultrasound Engine: A beamformer unit that processes raw ultrasound signals and optimizes image quality.
3. Display Device: A portable tablet PC that provides real‐time visualization and control of the ultrasound images.

The probe connects directly to the ultrasound engine, which is linked to the display device via a USB 3.0 cable. This configuration ensures seamless integration and high‐quality imaging while maintaining portability and usability.

### 2.8 Ergonomic and User‐Friendly Design

The SIBUS‐IN is designed to replicate the tactile sensation of traditional injection techniques, preserving the practitioner's ability to palpate and manipulate tissues. Its lightweight, curved design adapts to convex and concave anatomical regions, ensuring smooth movements and precise control. The device's intuitive interface and rapid learning curve make it accessible to practitioners with varying levels of ultrasound experience.

## 3 Discussion

The introduction of the SIBUS‐IN represents a pivotal development in aesthetic medicine, addressing key limitations of traditional ultrasound systems and contributing to improved standards in procedural safety and efficiency. Unlike conventional devices, which are often bulky and challenging to maneuver, the fingertip‐mounted design of the SIBUS‐IN allows enhanced precision and ease of use during aesthetic procedures. This ergonomic innovation significantly reduces practitioner fatigue and enhances access to anatomically complex regions, such as concave and convex facial structures.

The integration of advanced imaging capabilities further solidifies the SIBUS‐IN as a valuable tool. The high‐resolution 15 MHz probe delivers clear visualization, enabling practitioners to visualize skin layers, vascular networks, and deeper tissue structures in real time. The addition of advanced color imaging enhances the delineation of vascular structures, offering critical insights during high‐risk procedures and ensuring safer outcomes for patients. This feature is particularly advantageous when performing injectable treatments in areas with dense vascular anatomy, such as the nasolabial folds and periorbital regions.

While competing devices like the Clarius L20 HD and Mindray TE7 excel in providing generalized imaging solutions, they lack the specialized features required for dynamic aesthetic applications. For instance, these devices often necessitate two‐handed operation or pre‐procedural imaging, limiting their intra‐procedural utility [11]. The SIBUS‐IN, in contrast, integrates seamlessly into the practitioner's workflow, offering single‐handed operation and real‐time imaging that enhances procedural efficiency without compromising precision [12]. Additionally, the SIBUS‐IN replicates the tactile sensation of traditional injection techniques, preserving the practitioner's ability to palpate and manipulate tissues. This feature bridges the gap between conventional and technology‐assisted approaches, making the device accessible to both experienced professionals and those new to ultrasound‐guided aesthetics [12].

The inclusion of intuitive controls such as motion gesture recognition and voice activation demonstrates the forward‐thinking design philosophy underpinning the SIBUS‐IN. These features not only streamline procedural workflows but also maintain sterility by minimizing the need for physical adjustments during procedures. Furthermore, the wireless functionality eliminates the constraints of cumbersome cables, allowing for greater mobility and adaptability in diverse clinical environments.

The impact of the SIBUS‐IN extends beyond individual procedures to broader clinical practice. By enabling precise, real‐time visualization, the device contributes to improved patient outcomes and increased practitioner confidence. For example, during dermal filler injections, the SIBUS‐IN allows practitioners to visualize and avoid vascular structures, reducing the risk of complications such as vascular occlusion and tissue necrosis. In hyaluronidase injections for filler dissolution, the device enables precise targeting of filler material, minimizing collateral tissue damage. The ability to document and store imaging records also facilitates post‐procedural assessments and enhances medico‐legal compliance, further underscoring the device's value in modern aesthetic medicine.

The SIBUS‐IN also addresses several limitations of traditional ultrasound devices while presenting specific challenges that warrant consideration. The device's reliance on wireless connectivity may pose difficulties in environments with inadequate network infrastructure, while image quality can vary depending on facial anatomical complexities, particularly in highly curved contours and fibrous zones. This variability may affect the visualization of critical vascular structures, especially when combined with differences in operator technique and experience levels. Despite its intuitive interface, the system presents a learning curve for practitioners without prior ultrasound experience, as interpretation of sonographic images requires substantial training to differentiate between vascular and adjacent anatomical structures. Additionally, wearing an ultrasonographic device on the finger introduces potential infection concerns during aseptic procedures. These concerns could be mitigated through the use of disposable sterile covers specifically designed for the transducer, implementation of standardized decontamination protocols between patients, and potentially the development of single‐use components for high‐risk procedures. Sterile draping techniques extending beyond the device to include the practitioner's hand may further reduce contamination risks in aesthetic settings.

While the device shows promising features designed to enhance procedural safety and efficacy, comprehensive clinical validation through controlled studies remains necessary. Future research should focus on quantitative assessment of outcomes, including procedural efficiency and learning curve parameters across diverse practitioner groups and patient populations. Comparative studies with established ultrasound systems would further elucidate the relative advantages and limitations of this novel technology in real‐world clinical settings.

As the aesthetic ultrasound market continues to expand, the SIBUS‐IN is well‐positioned to address the growing demand for safe and efficient injectable treatments. Its tailored features and innovative design make it a compelling choice for aesthetic practitioners seeking to improve their standards of care. Future developments, such as the integration of artificial intelligence and machine learning models, aim to further enhance imaging capabilities, enabling automated guidance and improved diagnostic accuracy. Rather than replacing existing technologies, the SIBUS‐IN complements them by offering a specialized solution that aligns with the unique demands of aesthetic procedures [13].

## 4 Conclusion

The SIBUS‐IN fingerheld ultrasound machine represents a notable innovation in aesthetic medicine. By addressing the limitations of traditional devices and offering a compact, intuitive solution, it empowers healthcare professionals to deliver safer, more precise treatments. Its high‐quality imaging, ergonomic design, and seamless integration into standard practices mark an advancement in procedural safety and efficiency. As the demand for minimally invasive procedures continues to rise, the SIBUS‐IN is poised to become an valuable tool in the aesthetic practitioner's repertoire. Future advancements, including AI integration and expanded Doppler functionalities, will further solidify its role as a contributor to aesthetic ultrasound technology.

## Author Contributions

Conceptualization: Jovian Wan, Benjamin Ascher, Leonie Schelke, Isaac Kai Jie Wong, Peter J. Velthuis, Won Lee, Hee‐Jin Kim, Kyu‐Ho Yi. Writing – original draft preparation: Jovian Wan, Benjamin Ascher, Kyu‐Ho Yi. Writing – review and editing: Jovian Wan, Benjamin Ascher, Leonie Schelke, Isaac Kai Jie Wong, Peter J. Velthuis, Won Lee, Hee‐Jin Kim. Visualization: Jovian Wan, Benjamin Ascher, Leonie Schelke, Isaac Kai Jie Wong, Peter J. Velthuis, Won Lee, Hee‐Jin Kim, Kyu‐Ho Yi. Supervision: Kyu‐Ho Yi. All authors have reviewed and approved the article for submission.

## Disclosure

Dr. Benjamin Ascher is affiliated with Think‐In Tech, Paris, France, the developer of the SIBUS‐IN ultrasound device discussed in this manuscript. Dr. Ascher has contributed to the development of the product. This disclosure is provided to ensure transparency regarding any potential conflicts of interest. This study was conducted in compliance with the principles set forth in the Declaration of Helsinki.

## Conflicts of Interest

The authors declare no conflicts of interest.

## Supporting information


**Video S1.** The video demonstrates the use of the SIBUS‐IN fingerheld ultrasound machine during a facial injection procedure.

## Data Availability

The authors have nothing to report.
